# Detecting long tandem duplications in genomic sequences

**DOI:** 10.1186/1471-2105-13-83

**Published:** 2012-05-08

**Authors:** Eric Audemard, Thomas Schiex, Thomas Faraut

**Affiliations:** 1 Unité de Biométrie et Intelligence Artificielle, UR 875, INRA, Toulouse, France; 2 Laboratoire de Génétique Cellulaire, INRA, Toulouse, France

## Abstract

**Background:**

Detecting duplication segments within completely sequenced genomes provides valuable information to address genome evolution and in particular the important question of the emergence of novel functions. The usual approach to gene duplication detection, based on all-pairs protein gene comparisons, provides only a restricted view of duplication.

**Results:**

In this paper, we introduce ReD Tandem, a software using a flow based chaining algorithm targeted at detecting tandem duplication arrays of moderate to longer length regions, with possibly locally weak similarities, directly at the DNA level. On the *A. thaliana genome*, using a reference set of tandem duplicated genes built using TAIR,^a^ we show that ReD Tandem is able to predict a large fraction of recently duplicated genes (*dS* < 1) and that it is also able to predict tandem duplications involving non coding elements such as pseudo-genes or RNA genes.

**Conclusions:**

ReD Tandem allows to identify large tandem duplications without any annotation, leading to agnostic identification of tandem duplications. This approach nicely complements the usual protein gene based which ignores duplications involving non coding regions. It is however inherently restricted to relatively recent duplications. By recovering otherwise ignored events, ReD Tandem gives a more comprehensive view of existing evolutionary processes and may also allow to improve existing annotations.

## Background

Gene duplication has long been recognized as a major driving force in evolution. Both the extent of gene duplications in genomes and the theoretical formalization describing the process by which duplicate genes may contribute to genetic novelty by neo-functionalization lead to an intense interest for the subject (reviewed in [1-3]). The recent discovery of a previously unexpected dynamic of gene family expansion and contraction observed in complete genome sequences has called new attention on the phenomenon of gene duplication [4,5]. Moreover, recent studies of gene copy-number polymorphism in various organisms provide evidence of an ongoing mechanism of gene duplication and loss within species [6]. The different studies underline that this “revolving door” of gene gain and loss largely contributes to intra and interspecific phenotypic variability [7-9] and is therefore likely to have played an important role in shaping phenotypic differences among species [5].

Analysis of the genomes of *Arabidopsis*, human, mouse and rat revealed that tandemly arrayed duplicates account from 10% to 20% of all genes [2,10,11]. In addition, the contribution of tandem duplication to gene duplicates ranges from one-third in mammals [11] to almost 70% in *Caenorhabditis elegans*[12], highlighting the predominant role that tandem duplication plays in gene duplication. Tandem duplication contributes also to the evolution of other classes of functional elements such as exons within genes [13] or RNA genes [14]. In this respect, the detection of recent tandemly duplicated segments in complete genome sequences is a question of foremost interest.

Tandem duplication has been extensively studied at the protein coding gene level [11,15-17] or at the much smaller scale of serial repeats (micro-satellites), based on local DNA similarities [18,19].

All studies based on protein similarity analysis are naturally biased by the available genome annotation. In addition, such analyses automatically exclude duplicated segments with RNA genes or degenerated copies from the scope of the study. Despons and colleagues [20] have recently proposed an approach combining protein and DNA sequence comparison, enabling to detect degenerated paralogous copies, but the method still relies on an existing annotation and is additionally, as acknowledged by the authors, essentially limited to the analysis of compact genomes. Using DNA sequence comparison only, Eichler and colleagues [21,22] have significantly contributed to the understanding of dynamics of duplication in primates by studying highly identical duplicated DNA fragments greater than 1Kb, termed segmental duplications. This latter work is however limited to the study of very recent duplications.

On the other side of the size spectrum, different algorithms have been devised to detect so-called serial repeats at the DNA level. Initially targeted at short (micro-satellite-like) repeats, these algorithms have been considerably improved, leading to tools such as TRF [18] or mreps [19] which are capable detecting short tandem repeats on whole genomes. But, as shown in our experiments, the underlying definition of a serial repeat (as a contiguously repeated string) is not suitable for detecting large duplications that may contain disrupted similarities and which, despite being close, are far from contiguous.

**Figure 1:**
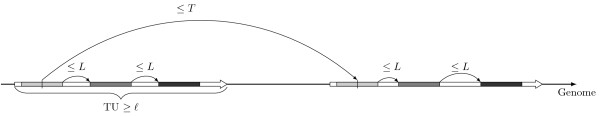
**Structure of detected Tandem Arrays.** An abstract representation of the structure of a Tandem Array with two Tandem Units that could be detected by Red Tandem. Every Tandem Unit has a minimum length ℓ and is separated from other Tandem Units in the array by less than *T* bases. Alignable units are reconstructed as a sequence of short similar segments (anchors) separated by less than *L* bases. In the *Arabidopsis thaliana* evaluation, we used ℓ = 500*bp*, *T* = 150*kb* and *L* = 40*kb*.

Despite the fundamental role of tandem duplication of large DNA fragments in the process of duplication-driven evolution, there is no existing method nor software to detect all identifiable tandemly duplicated segments from a DNA sequence. In principle, these tandemly duplicated segments could be any paralogous DNA segments that are tightly clustered on a chromosome. We propose the operational definition of tandemly duplicated segments as alignable, in a sense described below, paralogous segments with a minimum length of ℓ and with adjacent copies separated by a maximum distance *T* (see Figure 1).

In this paper, we introduce ReD Tandem, a tandem duplication detection tool that works from the genomic DNA sequence of the considered organism. In order to identify tandem duplicated segments, we start from short similar regions (also called anchors) that have been detected by a fast whole genome self-alignment software. These anchors are then chained into larger (duplicated) segments, similarly to what is done in synteny or segmental duplication detection tools such as DAGchainer [23] or OSfinder [24], modified to account for the specific properties of tandem duplications. In the next step, we analyse these chains to find tandem regions and an associated duplication unit. This duplication unit is used as a seed to locate further tandem duplications defining what we call a Tandem Array (TA).

In the first section, we present the formal definition of anchors and chains and the algorithm that enables to detect tandemly duplicated regions and the associated duplication unit. We next apply our method on *Arabidopsis thaliana* and we show that a large number of Tandem Gene Arrays, that can be derived from a CDS based family analysis, are detected by ReD Tandem. We analyse how the detection sensitivity varies with the evolutionary distance between genes. Finally we discuss the ability of the agnostic approach of ReD Tandem to detect duplications of RNA genes, duplications families involving different functional categories such as protein-coding genes together with long non-coding RNAs, as well as duplications of unannotated regions.

## Results and discussion

Tandem duplications typically include several copies of the same sequence. In the usual situation, these duplications have been obfuscated by evolution, leaving only local similarities. In this section we show how a specific chaining algorithm (called ReD) can reconstruct sets of duplicated regions that can be further analyzed to identify Tandem Arrays and associated duplication units. To test this approach, we apply it on the *Arabidopsis thaliana* genome and analyze its performance on characterized fraction of tandem *coding gene* duplications. Finally, we also explore the non coding fraction of the predicted tandem duplicated regions and show that ReD is also able to discover duplicated regions involving pseudo-genes, small or long RNA genes and other specific regions in the *Arabidospis thaliana* genome.

### Algorithm

To properly identify tandem arrays and their associated duplication unit, we follow a multiple steps procedure which is succinctly described now and described in more detail in the “Methods” section.

In a first step, adjacent sequence similarities are identified. These local similarities are next chained to identify a set of pairwise duplicated regions that could belong to tandem duplications. In a third step, the resulting chains are used to identify regions that could define tandem arrays together with the corresponding duplication unit. In the final step, in each such region, this duplication unit is used as a seed to reconstruct the structure of the complete tandem array.

#### Anchors detection

**Figure 2:**
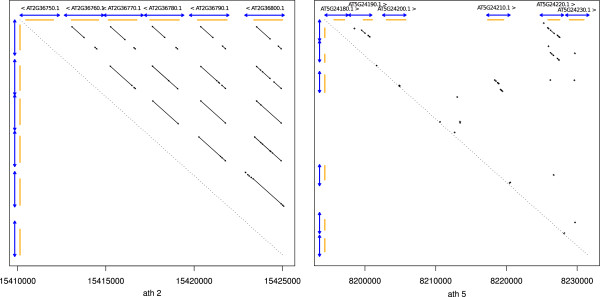
**Real tandem gene arrays, anchors and detection.** These two dotplots illustrate the detectable DNA similarities in two different regions carrying reference tandem gene arrays in the *Arabidopsis thaliana* genome. The small black diagonal arrows represent detected DNA anchors. The X and Y axis carry intervals representing TAIR10 annotated regions (in orange) and detected tandem units (in blue). The left region is a simple situation, with a recent series of duplications with clear anchors, easily analysed by ReD Tandem. In the right region, local similarities become rare and weak, defining a more complex pattern, which is again deciphered by ReD Tandem.

Given an initial DNA sequence, the analysis starts with the identification of all local self similarities, called “anchors” inside the sequence. Because of the specific situation of sequence self-alignment where self-overla pping alignments should be proscribed (see below and in the “Methods” section), we adapted an alignment program developed by one of the authors (glint, Faraut T,Courcelle E., unpublished) for this purpose. Each anchor *a* = (*a*_0_, *a*_1_) relates two regions of the genome. The first region *a*_0_, is assumed, for simplicity, to be on the forward strand. Dotplots offer a simple representation of a set of anchors (See Figure 2).

#### Anchors chaining

Similarly to what has been done for the reconstruction of homologous regions [23,24] or whole-genome alignment [25], our aim is to reconstruct duplicated regions as *consistently ordered* sequences of *close* anchors. By *consistent order*, we mean that each of the two sequences of regions defined by the sequence of anchors is either increasing (on the forward strand) or decreasing (on the reverse strand). To characterize *close anchors*, we use a distance introduced in [26] and defined in the “Methods” section.

The usual approach to identify large pairwise duplicated regions is to build a graph whose vertices are the detected anchors and where a directed edge (*ab*) is created when *a* and *b* are consistently ordered and sufficiently close to each other. A score is affected to the nodes and edges, reflecting the alignment score and the physical distances between anchors, and a minimum cost (shortest) path in this graph defines the regions sought [25].

By repeatedly extracting shortest paths from this acyclic graph, denoted as *G*_1_, one obtains a set of predicted duplicated regions, called chains, with an *overall cost* defined as the sum of the costs of all its paths.

**Figure 3:**
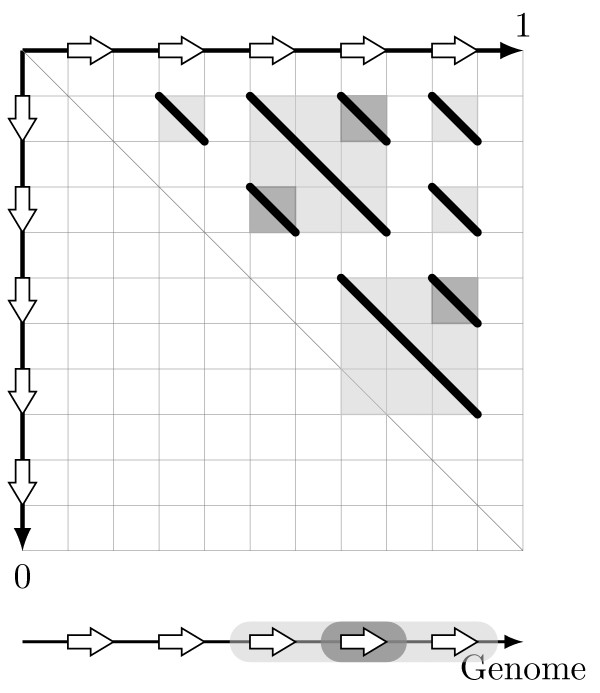
**Inconsistent chains.** A dotplot representing a set of chains (black lines) violating required properties for consistent sets of *t*-chains. The square under each chain capture the two intervals defined by the chain. When two squares overlap, property (4) is violated since intervals overlap. When a square meets the central dotplot diagonal, property (2) is violated.

Tandem arrays however present specific properties: they typically contain several close duplicated regions that can not overlap, which means (1) that the two regions defined by a chain should not overlap and (2) that for any pair of predicted chains, either their first regions do not overlap or their second regions do not overlap. These conditions are illustrated in Figure 3 and described in more detail in the “Methods” section.

Enforcing conditions (1) and (2) is difficult, especially because condition (2) is a global condition on the set of predicted chains and not only on each chain. Instead of using the usual process of repeated extraction of shortest chains, we therefore shift to a more sophisticated minimum cost flow based algorithm that will be able to produce a set of *k* chains that satisfy the constraints above and minimize their overall cost.

To use flows, the previous graph *G*_1_ is transformed in a transportation network and the problem of identifying a set of chains representing duplicated regions is reformulated into a minimum cost flow problem (see Methods).

Compared to the usual iterated greedy shortest path approach, this approach is able to “reconsider” previous chains and reallocate an anchor that was previously used in a chain to a new chain if this is needed to get an overall optimal cost. It therefore has a global view on the set of predicted chains. By iterating until (1) and (2) are satisfied we guarantee that the set of predicted chains are “non-overlapping” chains.

#### Tandem array and duplication unit identification

In order to delineate tandem array regions from the previous set of chains, we build a second graph *G*_2_ whose vertices are the predicted chains and remaining anchors and where two vertices are connected iff the associated regions of the two vertices on the genome overlap sufficiently on one of their sub-regions, *i.e.* the two chains/anchors share at least one sub-region.

Every connected component of this graph collect regions that share paralogous relationship and defines therefore a predicted tandem array region. The actual predicted tandem region is obtained by extending the minimum and maximum coordinates inside the connected component by a small margin. Every chain inside the connected component is a duplication unit candidate and the longest of all minimum cost chains in the array is used to identify the *reference duplication unit* (see Methods).

#### Final reconstruction

In order to increase sensitivity, the previously identified duplication unit is used as the query sequence in a TBLASTX [27] search against the tandem array region. All the candidate regions that align with the duplication unit on a sufficient length are kept as additional occurrences of the duplication unit and define the output of the prediction by the global “ReD Tandem” approach.

### Testing

The evaluation of the proposed method was performed using the *Arabidopsis thaliana* genome sequence as a test case. This genome and its internal gene duplications have been extensively studied providing an excellent standard for evaluation [28,29]. We used NCBI build 9.1, preprocessed using the low complexity filter DUST (Tatusov and Lipman, unpublished; described in [30]), anchors are produced using our own genome wide aligner, glint (Faraut T, Courcelle E., unpublished), using standard alignment scoring scheme (match +1, mismatch −3, gap open/extend −5/-2). Our aim is to test if tandem duplications identified by similarities between protein sequences can be recovered by ReD Tandem using the DNA sequence only. The first step therefore involves the construction of a reference set of tandem gene arrays against which the tandem duplications detected by ReD will be compared.

### Creating a reference set of tandem gene arrays

In order to construct a reference set of tandem gene arrays we proceed essentially like other published methods [10,20,31]. Considering the TAIR10 version of the *Arabidopsis thaliana* genome annotation, for each coding gene of length > 500 bp (matching the minimum length ℓ used in ReD Tandem), the longest annotated transcript is selected as the reference transcript. An all-against-all BLASTP comparison is conducted on the corresponding set of proteins. Two genes are considered to share a tandem paralogous relationship (resulting from an ancient tandem duplication) if they are less than *T* (150 kb) apart and exhibit a BLASTP hit with an e-value of at most 10^−5^ covering at least 70% of both sequences. These tandem paralogous relationships between genes are used in turn as anchors to create an overlap graph following the method described in “Methods”. Each connected component of this graph defines a tandem duplication array with genes as elementary duplication units. These connected components are essentially equivalent to the TGA defined in [10], with the difference that the notion of spacer genes between duplicated copies is replaced here by the physical distance threshold of *T* between copies to enable the comparison with our annotation-free approach.

These reference tandem duplication arrays will be called *tandem gene array*s (TGA), and the associated duplication unit *tandem gene unit* (TGU). Conversely, the regions and units detected by ReD Tandem from DNA alone will be respectively denoted as *tandem arrays* (TA), and associated *tandem unit* (TU).

### Evaluation criteria

In our analysis, we consider that a TGU is *detected* if it is overlapped on at least 70% of its length by a ReD TU. For TA and TGA, the criteria is more stringent and requires that the TGA is overlapped by more than 70% by the TA and that at least one of the TGU in the TGA is detected as a TU.

### Scanning Arabidopsis thaliana genome

From 60,021 DNA anchors, ReD Tandem built 10,290 chains with a mean of 2.9 anchors per chain, underlining the importance of chaining here. These chains define 1,718 *Tandem Array* (TA) covering 28.8% of the *A. thaliana* genome, made up of 5,477 *Tandem Unit* (TU) covering 10.6% of the sequenced genome. This is consistent with the estimated 10% of tandem gene duplications in the *Arabidopsis* genome [10].

### Comparison with the *reference set*: sensibility

We compared the results of ReD with the *reference set* to evaluate its sensitivity. The sensitivity is defined as the percentage of elements of the *reference set* which are *detected* by predictions of ReD Tandem. Results are given in Table 1. Overall, with 10.6% of the genome covered by TUs, ≃ 68% of all TGUs are detected.

**Table 1:** Sensitivity

Arabidopsis vs Arabidopsis	Total	Detected	%
TGA	1361	940	69
TGU	3694	2526	68.4

The table below gives the total number of TGA and TGU and the corresponding number and fraction of detected regions. A region is considered as detected if it is both overlapped by a TA on more than 70% of its length and one of its TGU has been detected. More than two thirds of all TGAs are detected. The fact that all TGUs cannot be detected is not surprising given that the oldest duplications cannot be detected at the DNA level.

**Figure 4:**
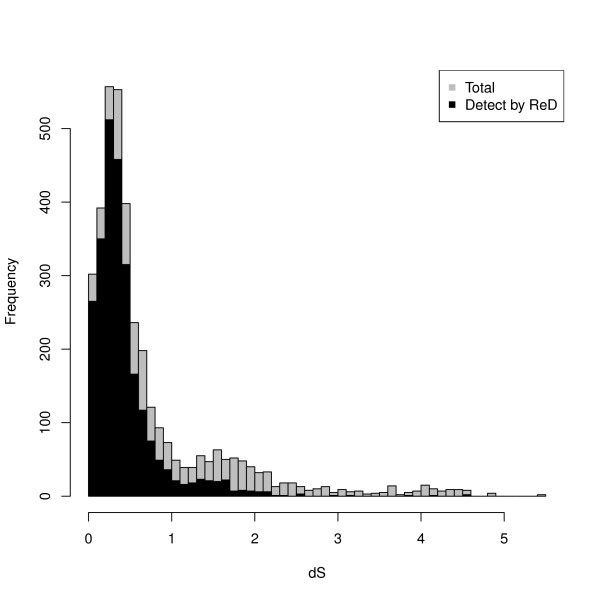
**Influence of duplication age on sensitivity.** This histogram shows the distribution of tandem gene unit pairs (in grey) and the associated proportion of tandem unit pairs detected by ReD Tandem (in black) as a function of the evolutionary distance as estimated by *dS*. As expected for a DNA based analysis, ReD Tandem is able to recover a large fraction of recently duplicated genes but is less efficient for older duplications.

**Figure 5:**
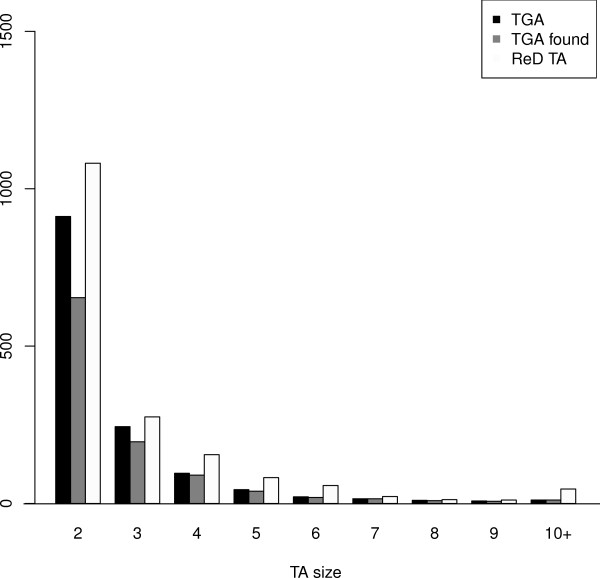
**TGA/TA size distributions.** Histogram showing the distribution of TGAs (black), detected TGAs (grey) and TAs (white) as a function of their size expressed in number of duplication units. Large TGAs are more easily detected than smaller ones.

**Figure 6:**
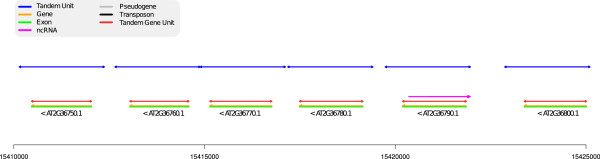
**A typical example of a detected TGA.** Region 15,410,000-15,425,000 of chromosome 2. Genes are indicated in green/yellow (exons/introns). Reference TGUs are indicated in red. The blue regions represent the detected TUs. The TGA is perfectly detected: each TU precisely corresponds to one TGU. The dotplot of this region is represented on the left of Figure 1. There are 666 TA similar to this one (all TUs detect TGUs of a TGA).

Since it relies only on DNA information, without annotations, the capacity of ReD Tandem to detect *Tandem Gene Array* (TGA) and *Tandem Gene Unit* (TGU) is influenced by the age of the duplication. The later has been measured using *dS* (number of silent substitutions) on tandem duplicated paralogous genes estimated using the method of Yang-Nielsen [32] as implemented in the PAML program [33]. Figure 4 shows how duplication age influences the detection power of our method. With a *dS* > 2, our algorithm hardly detects duplication unit. However, more than ≃ 79% of pairs of TGUs with *dS* ≤ 1 are detected (85% for *dS* < 0.5). Figure 5 shows the influence of family size on sensitivity. Most of the missing TGAs correspond to arrays with only two duplication units. Indeed, the score of such TGAs is shadowed by the extra bonus given to highly duplicated units (see Methods). These results however show that ReD Tandem can effectively detect tandem duplicated regions, at least when traces of the duplication are still observable at the DNA level. To give more flesh to these numbers, Figure 6 gives a typical example of a perfectly detected TGA with six TUs.

The results we have obtained on *A. thaliana* show that ReD Tandem, without relying on a predicted proteome, is able to correctly detect a large fraction of reference tandem duplicated genes provided they are sufficiently close from an evolutionary point of view (*dS* < 1).

The real added value of ReD Tandem is precisely its ability to perform its analyzes purely from DNA. Although it is restricted to “recent” duplications, ReD Tandem has the ability to identify duplicated regions which are implicitly censored by pure proteome based approaches, therefore helping to analyze the evolutionary history of the region. The only existing software that we know that provides related capabilities is [20] which uses BLASTX comparison in the immediate vicinity of every gene to identify possible pseudo-genes and gene relics. This approach, while still depending on an annotation, is, as acknowledged by the authors, essentially restricted to compact (bacterial or unicellular eukaryote) genomes. Because it relies on a direct comparison of the genome vs. itself that can be achieved using fast whole genome index based software, ReD Tandem is not restricted to the analysis of compact genomes. Serial repeat finders such as TRF or Mreps are also able to deal directly with DNA sequences, including large genomic sequences, but are instead restricted by the underlying definition of serial repeats (as contiguous repeats). To verify this, we applied both TRF (with default parameters) and Mreps (with a resolution of 50 allowing for maximum approximate matching) to the *A. thaliana* genome. TRF and Mreps identified respectively 35 and 26 serial repeats containing duplication units with a size above 500 bp (data not shown), compared to the 1,718 TA identified by ReD Tandem. By chaining local similarities, ReD Tandem is instead able to reconstruct large duplicated regions that may be interrupted by local loss of similarities.

Among the 5,477 TU predicted by ReD Tandem, around half of them correspond to protein genes. This leaves a large number of TUs essentially unknown in nature. To try to better understand the contents of these extra TUs, we compared them to the TAIR annotation of the genome (TAIR10) to evaluate if other annotated elements could be present in TUs. This comparison is presented in Table 2. We observe that TUs are more specifically enriched in pseudo-genes and pre-tRNA genes (which often appear clustered). To give some flesh to this table, we now give illustrative examples of various situations involving either non coding or unannotated regions.

**Table 2:** Comparison with annotated elements

Arabidopsis vs Arabidopsis	Total	Detected	Detected (%)
Gene	27169	3462	12.7
Trans. Element gene	3899	118	3.0
Pseudogene	871	220	25.2
Unknown gene	23	3	13.0
pre-tRNA	631	120	19.0
miRNA	174	19	10.9
snoRNA	71	8	11.3
Other RNA	301	29	9.6

The different types of annotated elements detected by TUs. The first column gives the number of annotated elements of each type in TAIR10. The second column gives the number of such element that are covered by TUs. The detected percentage of regions is indicated in the last column. The predicted TUs are enriched in pseudo-genes and pre-tRNA which often appear in clusters.

### Pseudogenization

**Figure 7:**
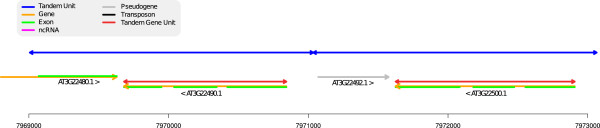
**An example of hybrid coding/non coding TA.** Region 7,969,000-7,973,000 of chromosome 3. A TA with two TUs. The first detected Tandem Unit covers the 3′ extremity of a coding gene and a complete coding gene. In the second unit, the complete gene is still there, but the 3′ partial gene has apparently lost his function and has been annotated as a pseudo-gene.

It has been widely accepted, for a long time, that pseudogenization is the most probable fate for duplicate coding gene copies, leading ultimately to gene relics [34]. In Figure 7, we give an example of a detected duplicated region containing annotated genes and one pseudo-gene. Here, the first duplicated region contains a complete gene and the partial 3′ extremity of a coding gene (AT3G22480). If the complete gene still appears as a gene in the second copy, the partial gene has, unsurprisingly, turned into the pseudo-gene AT3G22492.

### Gene fusion

**Figure 8:**
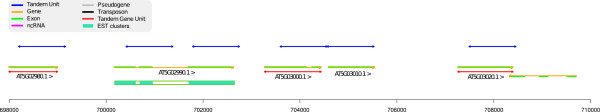
**An example of duplicated gene fusion.** Region 698,000-710,000 of chromosome 5. In this TA, four TUs match coding genes. The two remaining TUs appear each in a single fusion gene that has apparently been built by merging successive duplication units. The *A. thaliana* EST clusters (extracted from Gramene web site, Ensembl Plants Arabidopsis thaliana version 62.10), mapped to the region on the Gramene web site, effectively confirm the existence of an intron in this gene connecting the two duplicated regions.

In Figure 8, a TA with six TUs is represented. Four TUs among the six cover one coding gene each. These four genes are annotated as galactose oxidase/kelch repeat proteins. The two remaining TUs do not cover (by more than 70%) any functional element. Instead, they appear inside a single protein coding genes which seems to be the result of a gene fusion. This gene is also annotated as a galactose oxidase/kelch repeat protein. Existing evidence (*A. thaliana* EST cluster alignments extracted from Gramene web site for this region) seems to indicate that this is a real fusion and not the result of a mis-annotation.

### RNA clusters

**Figure 9:**
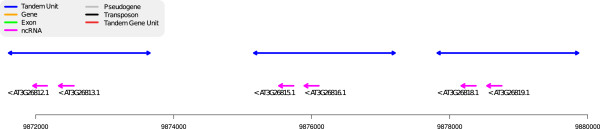
**An example of duplicated miRNA genes.** Region 9,872,000-9,880,000 of chromosome 3. A pure non coding TA where each TU (in blue) covers 2 miRNAs (in purple) and an upstream region.

A famous tandem duplication in the *A. thaliana* genome contains 81 tRNA genes in 27 tandem repetitions of a sequence containing three tRNAs (tRNA^*Tyr*^,tRNA^*Tyr*^,tRNA^*Ser*^[35]). This duplication is almost perfectly detected by ReD with 26 TU detected (Figure not shown, see http://narcisse.toulouse.inra.fr/ReDTandem/26.htmlath1-21268281-21308992). ReD actually detects several other RNA tandem duplications. As an example, we provide in Figure 9 an example of a detected tandem duplication with three copies of a pair of miRNAs.

#### lncRNA and CDS

**Figure 10:**
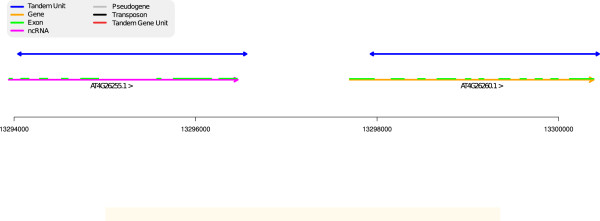
**A protein coding gene and a lncRNA.** Region 13,294,000-13,300,500 of chromosome 4. The two TUs of this TA respectively cover a lncRNA and a protein coding gene suggesting a possible protein-coding gene origin for the lncRNA.

The rich repertoire of long non coding RNAs has only been recently unveiled and little is known about their origin. Existing scenarios include both origination from scratch and transformation of protein genes into lncRNA [34]. The TA represented in Figure 10 shows two TUs matching respectively one lncRNA and one protein suggesting a possible protein-coding gene origin for this lncRNA gene. According to [34], such a metamorphosis has already been documented in mammals (Xist gene [36]) and Drosophilia. This region could be an example of a similar transformation in plants.

### Orphan TUs and TAs

These examples illustrate the fact that ReD Tandem ability to predict tandem duplications extends beyond pure tandem protein gene arrays. Still, TUs remain which do not cover any annotated element in *A. thaliana* genome. Among the 5,573 TUs predicted by ReD, 1,438 are orphan TUs. This is expected since ReD Tandem is just targeted at detecting DNA level tandem duplications. However, when such orphan TUs appear in a TA, other TUs in the same TA may provide extra information. Some of these orphan TUs may be of interest for improving the existing genome annotation.

**Figure 11:**
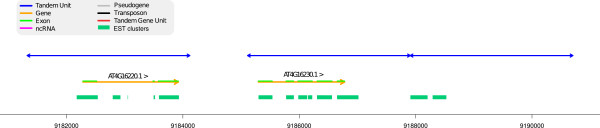
**A TU that could contribute to a modified annotation.** Region 9,181,000-9,191,000 of chromosome 4. In this TA, two TU match with coding genes but the third TU matches to no annotated element. However, there are EST matching in the 3rd region (extracted from Gramene web site, Ensembl Plants *Arabidopsis thaliana* version 62.10) and an associated FGENESH predicted gene (not shown), suggesting the existence of a 3rd transcribed region.

Figure 11 shows a TA where two TUs cover protein coding genes. The orphan TU on the right indicates a possibly missing gene (or pseudo-gene) in the annotation. This possibility is supported both by the existence of EST clusters alignments and associated FGENESH predictions in the region (extracted from Gramene web site).

We note in addition that ReD Tandem is also able to detect intertwined TGAs and to separate the duplication units belonging to the different families. (Figures not shown, see http://narcisse.toulouse.inra.fr/ReDTandem/6.htmlath4-8005015-8046308 and http://narcisse.toulouse.inra.fr/ReDTandem/2.htmlath4-8031970-8049154)

Finally, around a quarter (465 out of 1, 741) of all the TAs predicted by ReD Tandem are orphan TAs, that do not contain a single TU that covers or is covered by at least one TAIR10 annotation. These orphan TAs look genuinely different from the rest of all TAs in terms of TU size and number (see Table 3). The largest TA detected by ReD (with 70 TUs with a mean size around 600 bp, see http://narcisse.toulouse.inra.fr/ReDTandem/70.html) appears on chromosome 1:15088006 − 15430870. Interestingly, this region has been recently identified as a *CNV hotspot*[37].

**Table 3:** Orphan TAs

Arabidopsis vs Arabidopsis	Nb TAs	Nb TUs	Mean size of TU (bp)	Mean nb of TU
Orphan TAs	461	1504	1077	3.26
Other TA	1250	4068	2839	3.17

This table compares orphan TAs with other TAs in terms of their size (number of TUs and physical size). Orphan TAs tend have less and smaller TUs than the remaining TAs.

This section gives just a short extract of all detected TAs. A full list of detected TAs, with associated TUs and TAIR10 annotation for the *A. thaliana* genome with direct links to the corresponding region on Gramene web site is available from http://narcisse.toulouse.inra.fr/ReDTandem.

### Availability and requirements

The full packaged software from anchor detection to final TA/TU prediction is distributed under a CECILL open-source licence at http://narcisse.toulouse.inra.fr/ReDTandem. The software archive is also available as Additional file 1. You can either download a set of executable Linux 64 bits binaries wrapped in a Perl script or the set of sources. ReD Tandem is implemented in C++ and its execution time on *Arabidopsis thaliana* genome is around 4 hours on a single core computer. Its execution requires the availability of NCBI Blast and a Perl interpreter with the BioPerl package.

## Conclusions

In this paper we have introduced ReD Tandem, which, in our knowledge, is the first software targeted at predicting large partially conserved tandem duplications directly from DNA. This allows ReD Tandem to work directly on unannotated genomes. The analysis of ReD Tandem output and examples show that a pure DNA based analysis of tandem duplications unveils a large variety of phenomenons that cannot be revealed by usual protein based analysis. This uncensored vision of tandem duplication should be of great interest to address specific questions on duplication driven genome evolution such as the evolutionary fate of duplicated segments regarding their functional content [2,34].

From a pure evolutionary point of view, the Tandem Arrays and Tandem Units predicted by ReD Tandem and the usual protein gene based analysis [11,15,16,38] complement each other nicely. While a protein gene based analysis allows to identify distant evolutionary relationships, it implicitly censors all non coding elements that may be involved in the evolutionary process (pseudo-genes, gene relics, RNA genes, CNVs…). Conversely, we have shown that ReD Tandem is able to reliably detect relatively recent tandem duplications (*dS* < 1 typically) and can uncover a variety of duplications involving coding and non coding regions (and potentially totally non functional regions). It is therefore useful even if a current genome annotation exists and may help identify spurious or missing annotated elements. More importantly, it offers unprecedented direct raw access to tandem duplicated regions, directly bringing to light a variety of situations that were inaccessible in protein gene based approaches.

## Methods

### Preliminaries

The DNA sequence is modeled by a string *S*. A sub-string *u* of *S s*_*i*_…*s*_*j*_ will be simply noted as the interval [*i*, *j*] with a start *u*_*s*_ = *i* and an end *u*_*e*_ = *j*. For two non-overlapping intervals, *u* and *v*, *u* < *v* iff *u*_*e*_ < *v*_*s*_. If *u* < *v* we define $d\left(u,v\right)=v_{s}-u_{e}$.

A duplication copies a sub-string of *S* to a distinct location in *S* and a tandem duplication copies the original copy in its neighborhood. When the duplication is recent, the relationship between the original copy and the duplicate can be captured by a single sequence alignment.

### Anchors

Let *a* denote a local alignment (or *anchor*), between *S* and itself. *a* is a mapping between an interval $a^{0}=\left[a_{s}^{0},a_{e}^{0}\right]$ and another interval $a^{1}=\left[a_{s}^{1},a_{e}^{1}\right]$. Using the traditional 2-dimensional representation of an alignment - with *S* associated with the *x*-axis as well as the *y*-axis of the 2-dimensional plane $N^{2}$ - a local alignment is a path on the plane with $a^{0}=\left[a_{s}^{0},a_{e}^{0}\right]$ and $a^{1}=\left[a_{s}^{1},a_{e}^{1}\right]$ being the corresponding projections on the two axis, 0 standing for the *y*-axis and 1 for the *x*-axis. As usual, the orientation, or sign, of an anchor *a*, *a*.*sign*, indicates if the two aligned regions lie on the same strand (+) or not (−). We note *a*.*score* the alignment score of the anchor *a*. As anchors, defined by alignments, have often imprecise boundaries, when we compare two anchors we assume they are reduced to their mid-point.

Because of the symmetry in the comparison of *S* to itself, we can restrict ourselves to an upper-half-plane and impose, without loss of generality, that

(1) $$a_{s}^{0}<a_{s}^{1}$$

An anchor *a* which belongs to a genuine tandem duplication must satisfy some additional conditions: the two intervals *a*^0^ and *a*^1^ being intervals of *S*, as a consequence of the considered duplication mechanism, cannot overlap. In other words *a* cannot overlap itself on *S*. Together with (1), this therefore implies that *a*^0^ < *a*^1^. Since we consider only tandem duplications, *a*^0^ and *a*^1^ must be sufficiently close to each other on *S*. The two conditions can be formalized as follows

(2) $$\begin{array}{cc} \left(\text{i}\right) & a^{0}<a^{1}\\ \left(\text{ii}\right) & d\left(a^{0},a^{1}\right)\leq T \end{array}$$

where *T* is a user defined threshold. We note $d\left(a\right)=d\left(a^{0},a^{1}\right)$ the distance between *a*^0^ and *a*^1^, the two duplicated segments identified by the alignment *a*. Dot-plot examples of two contrasted real tandem arrays are illustrated in Figure 2.

When the duplication is a more ancient one, because of sequence divergence, the original region and the duplicate one can usually not be aligned on their full length. The identification of a duplication can be viewed as a special case of the genome alignment problem, a generalization of the sequence alignment problem. A common approach in genome alignment consists in chaining alignments [25]. A chain of anchors *c* is simply a path connecting anchors in the plane. This path relates the interval *c*^0^ on *S*, the projection of *c* on the *y*-axis and the interval *c*^1^ on *S*, the projection of *c* on the *x*-axis.

In order to build chains of anchors that reflect the proposed homology relationship between the two regions *c*^0^ and *c*^1^, we require the anchors *a*_1_, …, *a*_*n*_ in a chain to be co-linear: they must share the same sign and each sequence $a_{1}^{0},\ldots ,a_{n}^{0}$ and $a_{1}^{1},\ldots ,a_{n}^{1}$ must be totally ordered intervals on *S*. More formally we say that the anchor *b* can be a successor of anchor *a* in a chain, noted *a* ≺ *b*, if and only if

(3) $$\begin{array}{c} a.sign=b.sign\\ a^{0}<b^{0}\\ \left\{ \begin{array}{c} a^{1}<b^{1}\;\text{if}\;a.sign=+\\ a^{1}>b^{1}\;\text{if}\;a.sign=- \end{array}\right. \end{array}$$

The relation ≺ being a partial order on the set of anchors, it induces a directed acyclic graph where vertices are anchors and a directed edge (*a*_*i*_, *a*_*j*_) appears iff *a*_*i*_ ≺ *a*_*j*_. Any path in this graph is a chain of anchors. As a consequence of co-linearity (3), any chain inherits the shared sign of its anchors.

If furthermore the two intervals *c*^0^ and *c*^1^ defined by a chain *c* satisfy the properties (2), representing a candidate tandem duplication, we say that *c* is a *t*-chain. The purpose of the algorithm described below is to identify a set of *t*-chains in a sequence *S* and for each *t*-chain, identify the corresponding duplication unit, the region delineating the tandem array and the number of repetitions. Note that a *t*-chain can possibly be composed of a single anchor.

#### Identifying chains

In theory, anchors could be identified using any local self-alignment software (such as YASS [39]) but existing software usually do not produce anchors satisfying property (2). We therefore adapted our own genome-wide alignment software (glint, Faraut T, Courcelle E., unpublished) to this specific requirement. Optionally, the sequence can be preprocessed to deal with low complexity regions (see the Results section).

Starting from the DAG defined by the ≺ relation, we build a digraph by removing all edges (*ab*) that cannot participate in a *t*-chain, *i.e.* anchors *a* and *b* which define a chain with overlapping intervals or which are too distant (distance larger than *L*). If we note $\Delta^{0}=d\left(a_{i}^{0},a_{j}^{0}\right)$ and $\Delta^{1}=d\left(a_{i}^{1},a_{j}^{1}\right)$, following [26] we use

(4) $$d\left(a_{i},a_{j}\right)=2max\left\{\Delta^{0},\Delta^{1}\right\}-min\left\{\Delta^{0},\Delta^{1}\right\}$$

Compared to the Euclidian or Manhattan distances on the dotplot plan, this distance tends to be smaller when the closest extremities of two anchors lie on the same diagonal (with the same distance between their two intervals).

To identify the most likely set of *t*-chains in this graph, we will consider minimum cost paths in a graph weighted as follows:

· Every vertex *a*, representing an anchor, is weighted by its rescaled alignment score. For each position *i* of the sequence *S*, we define the coverage of nucleotide *s*_*i*_, *c(i)*, as the number of intervals *a*_._^0^, *a*_._^1^ containing the nucleotide *s*_*i*_.^b^ For each anchor *a*, *m*_*a*_ denotes the mean coverage of the associated intervals *a*^0^ and *a*^1^. The cost of vertex *a* in the anchor graph is defined by $-m_{a}\cdot a.score$, favoring the selection of anchors whose regions participate in other anchors.

· Every edge (*a*_*i*_, *a*_*j*_) connecting two anchors is initially weighted by the previous “distance” *d*(*a*, *b*) between anchors. To keep our algorithm efficient, we keep only the *k* best edges leaving every vertex (based on edge cost, typically *k* = 15). To normalize costs, edge score are rescaled so that the mean edge score is equal to the absolute value of the mean anchor score.

This defines the first digraph *G*_1_.

Importantly, since every *t*-chain represents a specific duplication event, two predicted *t*-chains *c*_*i*_ and *c*_*j*_ should not define overlapping intervals:

(5) $$either\{\begin{array}{c} c_{i}^{0}\cap c_{j}^{0}=\emptyset \\ c_{i}^{1}\cap c_{j}^{1}=\emptyset \end{array}$$

which also implies that *t*-chains cannot share anchors. Therefore, finding a set of *t*-chains with an *optimal global* score that satisfies properties (2), (3) and (4) cannot be simply computed by iteratively predicting successive *t*-chains using a traditional weighted chaining method [25]. Figure 3 shows a set of chains that would violate these properties.

To satisfy these properties, we transform the graph *G*_1_ in a transportation network [40] where edges and vertices are associated to unit capacity. All vertices are connected to a source and a sink with a unit capacity edge. Because vertices and edges have a unit capacity, any flow in this network defines a set of paths (chains) that, with guarantee, do not share any vertex (anchor).

In order to guarantee that this set of chains is a set of non overlapping *t*-chains satisfying a specific form of cost optimality, we use a variant of the successive shortest path algorithm for minimum cost maximum flow by Busaker and Gowen [40,41]. At iteration *i*, this algorithm provides a minimum cost flow of value *i*. This flow defines a set of *i* chains which do not share anchors with a global minimum cost (maximum score) among all such flows. If the set of chains defined shows no overlapping, we may stop. Otherwise, we proceed to the next iteration. It is easy to prove that the algorithm will terminate^c^ and therefore provides a set of *t*-chains *C*. This set has optimal cost among all sets of *t*-chains of same cardinality. These chains are the potential traces of locally duplicated regions that will be used in the next step to delineate tandem arrays.

#### Delineating potential tandem arrays: the overlap graph

Different chains in the set *C* may participate in the same tandem duplication. In order to delineate tandem duplicated regions, we build a new (undirected) graph *G*_2_, an overlap graph, whose vertices represent *t*-chains and remaining anchors. Two vertices are connected by an edge if the intervals they define overlap sufficiently on either axis. The connected components of this overlap graph define the tandem duplicated regions. More precisely the smallest interval encompassing all the projections of the anchors of a connected component defines the tandem duplicated region, or tandem array (TA), on *S* (in practice this interval is enlarged by *L* = 40 kb on each side).

For each TA, we try to infer the associated duplication unit from its *t*-chains. Because of the specific nature of tandem duplications, a single *t*-chain may contain multiple copies of the minimal duplication unit of the TA.^d^ To identify a minimal duplication unit, we start from the *t*-chain *c* with minimum cost (breaking ties with length). Its longest region is aligned against itself (with the alignment tool) and if less than 50% of the sequence is self-similar, we use it as the *reference duplication unit*. Otherwise, we trim the sequence from the extremity closest to the HSP with the smallest score and iterate.

### Final reconstruction

In order to improve the power of our algorithm to detect tandemly duplicated genes, the *reference duplication unit* is used as a TBLASTX query against the corresponding tandem region producing a new set of anchors. This new set of anchors is used to build a local weighted digraph *G*_3_. Since *G*_3_ only contains anchors involving the reference duplication unit, its structure is very simple. and a successive shortest path algorithm is used to extract the best chains, defining the detected tandem units (TU) of the TA. Ultimately, all detected TUs are enlarged by a maximum amount of 25% on each side, without violating constraints (4).

## Endnotes

^a^ The Arabidopsis Information Resource, at http://www. arbidopsis.org, centralizes information on the *A. thaliana* genome.

^b^ Note that because of property (2), for each anchor *a* at most one of the two intervals *a*^0^, *a*^1^ contains a nucleotide *s*_*i*_.

^c^ The maximum flow defines a set of chains of just one anchor therefore satisfying all conditions.

^d^ A tandem duplication with 4 duplication units can also be considered as a tandem duplication with 2 duplication units, each containing 2 smaller (minimal) units.

## Competing interests

The authors declare that they have no competing interests.

## Authors’ contributions

Algorithm design: EA, TS, TF. Coding, experimentation and evaluation: EA. Article drafting: TF,TS. All authors read and approved the final manuscript.

## Supplementary Material

Additional file 1 Archive containing the sources and licence for the ReD Tandem software. Click here for file
