# Supplementary material for: Detecting long tandem duplications in genomic sequences
Source: BMC Bioinformatics. 2012 May 8;13:83. doi: 10.1186/1471-2105-13-83 (PMC3464658; doi:10.1186/1471-2105-13-83)
Supplement: Additional file 1 — Archive containing the sources and licence for the ReD Tandem software. [file 1471-2105-13-83-S1.gz › ReDTandem-source/src/glint/license.html]

CeCILL FREE SOFTWARE LICENSE AGREEMENT


# CeCILL FREE SOFTWARE LICENSE AGREEMENT

## Notice

This
Agreement is a Free Software
license agreement that is the result of
discussions between its authors in order to ensure compliance with
the two main principles guiding its drafting:

- firstly,
  compliance with the principles governing the distribution of Free Software:
  access to source code, broad rights granted to users,
- secondly, the election of a governing law, French law, with which it is
  conformant, both as regards the law of torts and
  intellectual property law, and the protection that it offers to
  both authors and holders of the economic rights over software.

The
authors of the
CeCILL1
license are:

Commissariat
� l'Energie Atomique - CEA, a public
scientific, technical and industrial research establishment, having its
principal place of business at 25 rue Leblanc, immeuble Le Ponant D, 75015
Paris, France.

Centre
National de la Recherche Scientifique - CNRS,
a public scientific and technological establishment, having its
principal place of business at
3 rue Michel-Ange, 75794 Paris cedex 16, France.

Institut
National de Recherche en Informatique et en Automatique - INRIA,
a public scientific and technological establishment, having its
principal place of business at Domaine de Voluceau, Rocquencourt, BP
105, 78153 Le Chesnay cedex, France.

## Preamble

The
purpose of this Free Software
license
agreement
is to grant users
the right to modify and redistribute the software governed by this
license within the framework of an open source
distribution model.

The
exercising of these rights is conditional upon certain obligations
for users so as to preserve this status
for all subsequent
redistributions.

In consideration of access to
the source code and the rights to copy,
modify and redistribute granted by the license, users are provided only
with a limited warranty and the
software's author, the holder of the economic rights, and the
successive licensors only have limited liability.

In
this respect,
the risks
associated with loading, using, modifying and/or developing or
reproducing the software by the user
are brought to the user's attention,
given its Free Software
status,
which may make it
complicated to use,
with the result that its use is
reserved for developers and experienced professionals having in-depth
computer knowledge.
Users are therefore encouraged to load and test the suitability of the
software as regards their requirements in conditions enabling the security of
their systems and/or data to be ensured and, more generally, to use and
operate it in the same conditions of security. This Agreement may be freely
reproduced and published, provided it is not altered, and that no
provisions are either added or removed
herefrom.

This
Agreement may apply to any or all software for which the holder of
the economic rights decides to submit the use
thereof to its provisions.

## Article 1 - DEFINITIONS

For
the purpose of this Agreement,
when the following expressions
commence with a capital letter, they shall have the following
meaning:

Agreement:
means this license
agreement, and
its possible subsequent versions and annexes.

Software:
means the software in its Object Code and/or Source Code form and,
where applicable, its documentation, "as is"
when the Licensee accepts the Agreement.

Initial Software:
means the Software in its Source Code and
possibly its Object Code form and, where applicable, its
documentation, "as is" when it is
first distributed
under the terms and conditions of the
Agreement.

Modified Software:
means the Software modified by at least one Contribution.

Source Code:
means all the Software's instructions and program lines to which
access is required so as to modify the Software.

Object Code:
means the binary files originating from the
compilation of the Source Code.

Holder:
means the holder(s) of the economic rights over the Initial Software.

Licensee:
means
the Software user(s) having accepted the Agreement.

Contributor:
means a Licensee having made at least one
Contribution.

Licensor:
means the Holder, or any other individual or legal
entity, who distributes the Software under
the Agreement.

Contribution:
means any or all modifications,
corrections, translations, adaptations and/or new
functions
integrated into the Software by any or all Contributors,
as well as any or all
Internal Modules.

Module:
means a set of sources files including their documentation
that enables
supplementary
functions or services in
addition to those offered by the Software.

External Module:
means any or all Modules,
not derived from the Software,
so that this Module
and the Software run in
separate address spaces, with one calling the other when they are
run.

Internal Module:
means any or all
Module, connected to the Software so that they both execute
in the same address space.

GNU GPL:
means the GNU General Public License version 2 or any subsequent
version, as published by the Free Software Foundation Inc.

Parties:
mean both the Licensee and the Licensor.

These
expressions may be used both in singular and plural form.

## Article 2 - PURPOSE

The
purpose of the Agreement is the grant
by the Licensor to the
Licensee of a non-exclusive, transferable
and worldwide license
for the Software as set forth in Article 5
hereinafter for the whole term of the
protection
granted by the rights over said Software.

## Article 3 - ACCEPTANCE

3.1 The
Licensee shall be deemed as having accepted
the terms and conditions of this Agreement
upon the occurrence of the
first of the following events:

- (i)
  loading the Software by any or all means, notably, by downloading
  from a remote server, or by loading from a physical medium;
- (ii)
  the first time the Licensee exercises any of the rights granted
  hereunder.

3.2 One
copy of the Agreement, containing a notice relating to the
characteristics
of the Software, to the limited warranty, and to the
fact that its use is
restricted to
experienced users has been provided to the
Licensee prior to its acceptance as set forth in Article
3.1
hereinabove, and the Licensee hereby acknowledges that it
has read and understood it.

## Article 4 - EFFECTIVE DATE AND TERM

### 4.1 EFFECTIVE DATE

The
Agreement shall become effective on the date when it is accepted by
the Licensee as set forth in Article 3.1.

### 4.2 TERM

The
Agreement shall remain in force
for the entire legal term of
protection of the economic rights over the Software.

## Article 5 - SCOPE OF RIGHTS GRANTED

The
Licensor hereby grants to the Licensee, who
accepts, the
following rights over the Software
for any or all use, and for
the term of the Agreement, on the basis of the terms and conditions
set forth hereinafter.

Besides, if the Licensor owns or comes to
own one or more patents
protecting all or part of the
functions of the Software
or of its components,
the Licensor undertakes not to enforce the rights granted by these
patents against successive Licensees using, exploiting or
modifying the Software. If these patents are
transferred, the Licensor
undertakes to have the transferees subscribe to the
obligations set forth in this
paragraph.

### 5.1 RIGHT OF USE

The
Licensee is authorized to use the Software, without any limitation as
to its fields
of application, with it being hereinafter
specified that this comprises:

1. permanent
   or temporary reproduction of all or part of the Software by any or
   all means and in any or all form.
2. loading,
   displaying, running, or storing the Software on any or all
   medium.
3. entitlement
   to observe, study or test
   its operation
   so as to
   determine
   the ideas and principles behind any or all
   constituent elements of said Software. This shall apply when
   the
   Licensee carries out any or all loading, displaying, running,
   transmission or storage operation as regards the Software,
   that it is entitled to carry out hereunder.

### 5.2 ENTITLEMENT TO MAKE CONTRIBUTIONS

The
right to make Contributions includes the right to translate, adapt,
arrange, or make any or all modifications to the Software,
and the
right to reproduce the resulting software.

The
Licensee is authorized to make any or all Contributions to
the Software provided that it
includes an explicit notice that it is the
author
of said Contribution and indicates the date of the
creation thereof.

### 5.3 RIGHT OF DISTRIBUTION

In
particular, the right of distribution includes the
right to publish, transmit and communicate
the Software to the general public
on any or all medium, and by any or all means, and the right to
market, either in consideration of a fee, or free of charge,
one or more copies of the Software
by any
means.

The
Licensee is further authorized to
distribute copies of the
modified or unmodified Software to third parties according to the
terms and conditions set forth hereinafter.

#### 5.3.1 DISTRIBUTION OF SOFTWARE WITHOUT MODIFICATION

The
Licensee is authorized to distribute
true copies of the Software
in Source Code or Object Code form, provided that
said distribution
complies with all the provisions of the
Agreement and is accompanied by:

1. a
   copy of the Agreement,
2. a
   notice relating to the limitation of both the Licensor's
   warranty and liability as set forth in Articles 8 and 9,

and
that, in the event that only the Object Code
of the Software is redistributed, the Licensee
allows future Licensees unhindered access
to the
full Source Code of the Software
by indicating how to access it,
it being
understood that the additional cost of acquiring the Source
Code shall not exceed the cost of transferring the data.

#### 5.3.2 DISTRIBUTION OF MODIFIED SOFTWARE

When
the Licensee makes a Contribution to the Software, the terms and
conditions for the distribution
of the resulting Modified Software become subject to all the provisions
of this Agreement.

The
Licensee is authorized to distribute
the Modified Software, in
source code or object code form, provided that said
distribution
complies with all the provisions of the Agreement and is accompanied
by:

1. a
   copy of the Agreement,
2. a
   notice relating to the limitation of both the Licensor's
   warranty and liability as set forth in Articles 8 and 9,

and
that, in the event that only the
object code of the Modified Software
is redistributed, the Licensee allows future Licensees
unhindered access to the
full source code of the Modified Software
by indicating how to access it,
it being understood that the additional cost of acquiring the source
code shall not exceed the cost of transferring the data.

#### 5.3.3 DISTRIBUTION OF EXTERNAL MODULES

When
the Licensee has developed
an External Module, the terms and
conditions
of this Agreement do not apply to said
External Module, that may be
distributed under a separate
license agreement.

#### 5.3.4 COMPATIBILITY WITH THE GNU GPL

The Licensee can include a code that is subject to the provisions
of one of the versions of the GNU GPL in the Modified or unmodified
Software, and distribute that entire code under the terms of the
same version of the GNU GPL.

The Licensee can include the Modified or unmodified Software in a
code that is subject to the provisions of one of the versions of
the GNU GPL, and distribute that entire code under the terms of
the same version of the GNU GPL.

## Article 6 - INTELLECTUAL PROPERTY

### 6.1 OVER THE INITIAL SOFTWARE

The
Holder owns the economic rights over the Initial Software. Any or
all use of the Initial Software is subject to compliance with the
terms and conditions under which the Holder has elected to
distribute its work and no one shall be entitled to modify the terms
and conditions for the distribution of said Initial Software.

The
Holder undertakes that the Initial
Software will remain ruled at least by this Agreement,
for the duration set
forth in Article 4.2.

### 6.2 OVER THE CONTRIBUTIONS

The Licensee who develops a Contribution is the owner
of the intellectual property rights over this Contribution
as defined by applicable law.

### 6.3 OVER THE EXTERNAL MODULES

The Licensee
who develops an External Module
is the owner of the intellectual property rights over
this External Module as defined by applicable law and is
free to choose the type of agreement that shall govern its
distribution.

### 6.4 JOINT PROVISIONS

The
Licensee expressly undertakes:

1. not to remove, or modify, in any manner, the
   intellectual property notices attached to the Software;
2. to reproduce said notices, in an identical manner, in the copies of
   the Software modified or
   not.

The
Licensee undertakes not to directly or indirectly infringe
the intellectual property rights of the Holder and/or Contributors
on the Software
and to take, where applicable, vis-�-vis its staff, any and
all measures required to ensure respect of
said intellectual
property rights of the Holder and/or Contributors.

## Article 7 - RELATED SERVICES

7.1 Under
no circumstances shall the Agreement oblige the Licensor to provide
technical assistance or maintenance services for the Software.

However,
the Licensor is entitled to offer this type of
services. The terms
and conditions of such technical assistance, and/or such
maintenance, shall be set forth in a separate
instrument. Only
the Licensor offering said maintenance and/or technical assistance
services shall incur liability therefor.

7.2 Similarly,
any Licensor is entitled to offer to its
licensees, under its
sole responsibility,
a warranty, that shall only
be binding upon itself, for the redistribution of the Software
and/or the Modified Software, under terms and conditions
that it is free to decide. Said warranty, and the financial
terms and conditions of its application, shall be subject
of a separate
instrument executed between the Licensor and the Licensee.

## Article 8 - LIABILITY

8.1 Subject
to the provisions of Article 8.2,
the Licensee shall be entitled to claim compensation for
any direct
loss it may have suffered from the Software
as a result of a fault on the part of the relevant
Licensor, subject to
providing evidence thereof.

8.2 The
Licensor's liability is limited to the commitments made under
this Agreement and shall not be incurred as a result of in
particular: (i) loss due the Licensee's total or partial
failure to fulfill its obligations, (ii) direct or consequential
loss that is suffered by the Licensee due to the
use or performance of the Software,
and (iii)
more generally, any
consequential loss.
In particular
the Parties expressly
agree that any or all pecuniary or business loss (i.e. loss of data,
loss of profits, operating loss, loss of customers or orders,
opportunity cost, any disturbance to business activities) or any or
all legal proceedings instituted against the Licensee by a third
party, shall constitute consequential loss and shall not provide
entitlement to any or all compensation from the Licensor.

## Article 9 - WARRANTY

9.1 The
Licensee acknowledges that the
scientific and technical
state-of-the-art when the Software
was
distributed did not enable all possible uses to be tested and
verified, nor for the presence of
possible defects to be detected.
In this respect, the Licensee's attention has been drawn to
the risks associated with loading, using, modifying and/or
developing and reproducing the Software
which are reserved for
experienced users.

The
Licensee shall be responsible for verifying, by any or all means,
the suitability of the product for its requirements, its good working
order, and for ensuring that it shall not cause damage
to either persons or
properties.

9.2 The Licensor
hereby represents, in good faith, that it is entitled to grant all
the rights over the Software (including in
particular the rights set
forth in Article 5).

9.3 
The
Licensee acknowledges that the Software is supplied "as is"
by the Licensor without any other express or tacit
warranty,
other than that provided for in Article
9.2 and, in particular,
without any warranty as to its
commercial value, its secured, safe,
innovative or relevant nature.

Specifically,
the Licensor does not warrant that the Software is free from any
error, that it will
operate without interruption,
that it will be
compatible with the Licensee's own equipment and
software configuration,
nor that it will meet the
Licensee's requirements.

9.4 The
Licensor does not either expressly or tacitly warrant that the
Software does not infringe any third party
intellectual
property right
relating to a patent, software or any
other property right.
Therefore, the Licensor disclaims any and all liability towards the
Licensee arising out of any or all proceedings for
infringement that may be
instituted in respect of the use, modification and redistribution of
the Software. Nevertheless, should such proceedings be instituted
against the Licensee, the Licensor shall provide it with technical
and legal assistance for its defense. Such technical and legal
assistance shall be decided on a case-by-case basis
between the
relevant Licensor and the Licensee pursuant to a memorandum of
understanding. The Licensor disclaims any and
all liability as
regards the Licensee's use of the
name of the Software. No
warranty is given as regards
the existence of prior rights
over the name of the Software or as regards
the existence of a trademark.

## Article 10 - TERMINATION

10.1 
In the event of a breach by the
Licensee of its obligations hereunder, the Licensor may
automatically terminate this Agreement thirty (30)
days after
notice has been sent to the Licensee and has remained ineffective.

10.2 A
Licensee whose Agreement is terminated shall no longer be authorized
to use, modify or distribute the Software. However, any
licenses that it may have granted prior to termination of the
Agreement shall remain valid subject to their having been granted in
compliance with the terms and conditions hereof.

## Article 11 - MISCELLANEOUS

### 11.1 EXCUSABLE EVENTS

Neither
Party shall be liable for any or all delay, or failure to perform
the Agreement, that may be attributable to an event of force
majeure, an act of God or an outside cause, such as
defective functioning or interruptions
of the electricity or
telecommunications networks, network
paralysis following a virus attack, intervention by
government authorities, natural disasters, water damage,
earthquakes, fire, explosions, strikes and labor unrest, war, etc.

11.2 Any failure by either
Party, on one or more
occasions, to invoke one or more of the
provisions hereof, shall under no
circumstances be interpreted as being a waiver by the interested
Party of its right to invoke said
provision(s) subsequently.

11.3  The
Agreement cancels and replaces
any or all previous agreements, whether written or oral,
between the
Parties and having the same purpose, and constitutes the entirety of
the agreement between said Parties concerning said purpose. No
supplement or modification to the terms and conditions hereof shall
be effective as between the Parties
unless it is made in writing and
signed by their duly authorized representatives.

11.4 In
the event that one or more
of the provisions hereof were to conflict with a current or future
applicable act or legislative text, said act or legislative text
shall prevail, and the Parties
shall make the necessary
amendments so as to comply with
said act or legislative
text. All other provisions shall remain
effective. Similarly,
invalidity of a provision of the
Agreement,
for any reason whatsoever, shall not cause the Agreement as a whole
to be invalid.

### 11.5 LANGUAGE

The
Agreement is drafted in both French and English
and both versions are
deemed authentic.

## Article 12 - NEW VERSIONS OF THE AGREEMENT

12.1 Any
person
is authorized to duplicate and distribute copies of
this Agreement.

12.2 So
as to ensure coherence, the wording of this Agreement is protected
and may only be modified by the authors of the License,
who reserve
the right to periodically publish updates or new versions of the
Agreement, each with a separate number. These subsequent versions
may
address new issues encountered by Free Software.

12.3 Any
Software distributed under a given version of the Agreement
may only be subsequently distributed under the same version of the
Agreement or a subsequent version, subject to the provisions of
Article 5.3.4.

## Article 13 - GOVERNING LAW AND JURISDICTION

13.1 
The Agreement is governed by French law. The
Parties agree to endeavor to seek an amicable
solution to any disagreements or disputes
that may arise during the performance of
the Agreement.

13.2 Failing an amicable solution
within two (2) months as from their
occurrence, and unless emergency proceedings are necessary, the
disagreements
or disputes shall be referred to the Paris Courts having
jurisdiction, by the more diligent
Party.

1 CeCILL stands for Ce(a) C(nrs) I(nria) L(ogiciel) L(ibre)

Version 2.0 dated 2006-09-05.
